# Cortisol, DHEA, and Sexual Steroid Concentrations in Fattening Pigs’ Hair

**DOI:** 10.3390/ani9060345

**Published:** 2019-06-12

**Authors:** Cristina Bergamin, Antonella Comin, Mirco Corazzin, Massimo Faustini, Tanja Peric, Annalisa Scollo, Flaviana Gottardo, Marta Montillo, Alberto Prandi

**Affiliations:** 1 Department of Agricultural, Food, Environmental and Animal Sciences, University of Udine, 33100 Udine, Italy; bergamin.cristina@spes.uniud.it (C.B.); antonella.comin@uniud.it (A.C.); mirco.corazzin@uniud.it (M.C.); marta.montillo@uniud.it (M.M.); alberto.prandi@uniud.it (A.P.); 2 Department of Veterinary Medicine, University of Milano, 20133 Milano, Italy; massimofaustini@gmail.com; 3 Department of Animal Medicine, Production and Health, University of Padova, 35020 Agripolis Legnaro (PD), Italy; scollo@suivet.it (A.S.); flaviana.gottardo@unipd.it (F.G.)

**Keywords:** fattening pigs, hair, steroid hormones, allostatic load

## Abstract

**Simple Summary:**

The rearing of heavy pigs in Italy is an important part of the production of Protected Designation of Origin (PDO) hams. Along with the standard quality characteristics, the quality of products with animal origin is also assessed by the level of animal welfare. Evaluation of hair steroid concentrations has been considered an effective approach to assess stress in mammals. The advantage of using hair for this process is that it provides an integrated measure of hormone concentrations over medium- and long-term periods, it can be simply and non-invasively collected, and it does not require any special expedient for storage. The aim of this study was to evaluate the hair cortisol, dehydroepiandrosterone (DHEA), and sexual steroid concentrations in fattening pigs at 36 weeks of age before slaughtering through a non-invasive approach. Females had significantly higher cortisol levels, significantly lower concentrations of DHEA, and significantly higher cortisol/DHEA ratios than barrows. Progesterone was significantly higher in gilts than in barrows. Testosterone and 17β-estradiol were significantly higher in barrows than in gilts. These results will allow us to plan future research with the aim of identifying threshold values in order to set up strategies to control the allostatic load and to increase the resilience of fattening pigs.

**Abstract:**

The aim of this study was to analyze the feasibility and reliability of using hair as a matrix to determine the dehydroepiandrosterone (DHEA) and sexual steroid concentrations and the cortisol/DHEA ratio in fattening pigs. The results could be also used to plan future research to identify threshold values in order to set up strategies to control the allostatic load and increase the resilience of fattening pigs before slaughter. The study was conducted on 107 commercial crossbred rearing pigs. The hair samples were taken by shaving at the age of 36 weeks, and concentrations of the hormones were measured using a solid-phase microtiter radioimmunoassay. Females had significantly higher cortisol levels (*p* < 0.01), significantly lower DHEA concentrations (*p* < 0.05) and significantly higher cortisol/DHEA ratios (*p* < 0.01) than barrows. Progesterone was significantly higher in gilts than in barrows (*p* < 0.01). Testosterone and 17β-estradiol were significantly higher in barrows than in gilts (*p* < 0.05). If future research can produce threshold values for the different markers examined, the evaluation of animals under subclinical stress conditions will be possible.

## 1. Introduction

The rearing of heavy pigs in Italy is an important part of the production of Protected Designation of Origin (PDO) hams [1], such as the “Prosciutto di San Daniele” and the “Prosciutto di Parma” (EU Council Regulation no. 510/2006). In order to maintain the appropriate qualitative characteristics of the PDO hams, some constraints are provided, i.e., the pigs must be at least 9 months old and have a live weight of 160 kg at slaughter. These apply to both barrows and gilts. Along with the standard quality characteristics, the quality of products with animal origin is also measured by accounting for animal welfare; it is also well-known that animal welfare is directly related to improved production efficiency [2].

Production systems for fattening pigs, particularly in large farms and group sizes, have been under serious discussion, mainly due to animal welfare concerns [3,4].

The assessment of the resilience and allostatic load of animals could be used to improve their welfare. Allostatic load and resilience can be evaluated through biological markers, such as cortisol, dehydroepiandrosterone (DHEA) [5,6,7], dehydroepiandrosterone sulfate (DHEA-S) [8], and their ratios [9,10]. Also, sex hormones such as progesterone (P4), 17β-estradiol (E2), and testosterone are involved in the stress response. A stressful living environment negatively influences reproductive performance [11], growth rate, and immunity [12] in animals when adverse environmental stressors exceed threshold limits for coping and compensatory mechanisms [13]. In fact, mammals normally attempt to respond to environmental, physical, and psychosocial challenges through a continuous evaluation of needs and the adaptation of physiologic set points [14]. 

Several matrices can be used to detect the concentrations of these markers: blood [15], saliva [16], urine [17], feces [18], claws [19,20], and hair [21,22,23]. Unlike the traditional matrices (serum, saliva, urine, or feces) that are subjected to major physiological daily fluctuations [15,24,25] and provide a measurement of the concentration at a single time point or within a 12–24 h period, the analysis of hair is unaffected by circadian variations in the hormone or by factors inducing short-term variations [26]. Furthermore, hair provides a retrospective calendar of exposure to hormones [26,27,28] and an integrated measure of hormone concentrations over medium- and long-term periods [24]. A single time point measurement cannot reflect the integral of systemic exposure. A similar approach, for example, is the use of the glycated hemoglobin assay for the diagnosis of diabetes.

Hair can be simply and non-invasively collected, and it does not require any special expedient for storage. As pointed out by Heimbürge et al. [29] in a review, the use of hair hormone analysis requires some recommendations, such as the use of animals with the same age, sex, and reproductive state; the sampling of hairs from the same body region and of the same color; the consideration of the time delay between hormone incorporation and sampling of hair that depends on hair growth velocity, which may vary between species and body regions; the avoidance of external contamination; and the use of the “shave–reshave” method, if possible, to establish a precise timeframe. If a non-regrowth hair sample is used, one must consider that the pig hair follicles pass through one complete cycle per year, that molting of the telogen follicles occurs during the summer, and that the seasonal changes have a marked effect on the activity of the hair follicle (maximum percentage of the anagen phase from August to January) [30,31].

The main objective of this study was to analyze the feasibility and reliability of using hair as a matrix to determine the DHEA and sexual steroid concentrations and the cortisol/DHEA ratio in fattening barrows and gilts that could be also used to plan future research to identify threshold values in order to set up strategies to control the allostatic load and increase the resilience of fattening piglets before slaughter.

## 2. Materials and Methods 

### 2.1. Ethics

Although hair sampling is non-invasive and it is not a troublesome procedure, the study was carried out in accordance with Directive 2010/63/EU on the protection of animals used for scientific purposes. Animals were reared according to the current Italian legislation which implements Council Directive 2008/120 EC, laying down minimum standards for the protection of pigs.

### 2.2. Animals and Housing Conditions

A group of 1800 Goland crossbreed pigs reared on one commercial farm in Northern Italy for PDO “Prosciutto di Parma” ham production [32] was initially considered.

The herd was part of a close-cycle pig holding facility, and the animals were born at the beginning of August. They were weaned at the age of 28 days, and then they were moved to the post-weaning facility until the beginning of the fattening phase that started at 12 weeks of age when the animals weighed between 45 and 55 kg. At the beginning of the fattening phase, 107 pigs (45 barrows and 52 gilts) of the same age from eleven pens (9–10 pigs/pen) were selected. Selection of the animals was made by expert technicians who excluded small, excessively heavy, and sick animals. Pigs were remixed only when they were moved from the post-weaning facilities to the fattening barns. The pigs were undocked, and the pens were partitioned such that the pigs could have visual and nose-to-nose contact between neighboring pens. The facility was equipped with an automatic control system for temperature and ventilation, and each pen had a computerized feeder and bowl-type drinking stations. The electronically controlled temperature was set at 22 °C at the beginning of the fattening cycle, was decreased to 20 °C after four weeks, and then maintained until the end of this phase. The stocking density was the same for each pen, and the area available for each pig was in accordance with Directive 2008/120/EC. Liquid feed was provided three times per day, and the food composition was consistent with the requirements of the PDO protocol [32].

### 2.3. Clinical Evaluation of Animals

Veterinary clinical evaluations were carried out by a highly qualified veterinary practitioner every two weeks, and the animals were deemed to be clinically healthy. No skin inflammation or lesions were recorded throughout the study period.

### 2.4. Hair Sampling

Hair samples (about 100–150 mg) were taken in May from the pigs at the age of 36 weeks. Animals were randomly selected from each pen and, after being gently restrained, hair samples were carefully obtained by shaving close to the skin with electric clippers on the dorsal area of the neck, behind the ears. The area was chosen because it is cleaner than other areas. The hair hormones deposited in the hair shaft can reflect a period from birth until the collection of the samples (36 weeks), with the exclusion of the last 15 days of each sampling period [30,31]. The shaved hair samples were stored in paper envelops and, after being individually marked, they were stored in the dark and at room temperature until analysis.

### 2.5. Hair Washing Procedure and Extraction

Hair samples were washed using the method of Bacci et al. [22]. Then, the samples were extracted according to the method described by Trevisan et al. [23]. In brief, 60 mg of trimmed hair was placed in a glass vial along with 3 mL of methanol. The vials were incubated at 37 °C for 16 h. Next, the liquid in the vial was evaporated to dryness at 37 °C under an airstream suction hood. The remaining residue was dissolved in 0.8 mL of 0.05 M phosphate-buffered saline (PBS) at pH 7.5.

### 2.6. Hair Hormone Analysis

The concentrations of cortisol, DHEA, P4, E2, and testosterone were measured using a solid-phase microtiter RIA (radioimmunoassay). In brief, a 96-well microtiter plate (OptiPlate; PerkinElmer Life Sciences Inc.) was coated with goat anti-rabbit γ-globulin serum diluted 1:1000 in 0.15 mM sodium acetate buffer (pH 9) and incubated overnight at 4 °C. The plate was then washed twice with RIA buffer (pH 7.4) and incubated overnight at 4 °C with 200 μL of the antibody serum diluted at ratios of 1:20,000 for cortisol (Analytical Antibodies, Bologna, Italy), 1:2000 for DHEA (Sigma-Aldrich, St. Louis, MO, USA), 1:8000 for P4 (Analytical Antibodies, Bologna, Italy), 1:80,000 for E2 (Analytical Antibodies, Bologna, Italy), and 1:160,000 for testosterone (Analytical Antibodies, Bologna, Italy). The cross-reactivities of the anti-cortisol antibody with other steroids were as follows: cortisol, 100%; corticosterone, 1.8%; aldosterone, <0.02%. The cross-reactivities of the anti-DHEA antibody with other steroids were as follows: DHEA, 100%; pregnenolone, 0.1%; androstenediol, 0.08%; dihydrotestosterone, 0.05%; sulfate DHEA, 0.02%; testosterone, 5α-androstane-diol-3β, 5α-androstane-diol-3α, androsterone, epiandrosterone, and 5β-androstane-3, 9.2%; epiandrosterone, 2.8%; pregnenolone, 1.8%; 5α-androstane-diol-3α, estradiol, progesterone, cholesterol, and estrone, <0.001%. The cross-reactivities of the anti-P4 antibody with other steroids were as follows: 11 β-OH-progesterone, 46%; 17 α-OH-progesterone, 0.4%; 20 α-OH-progesterone, 0.04%; testosterone, 0.08%; cortisol, <0.01%; 17β-estradiol, <0.01%; 17α-estradiol, <0.01%; estrone, <0.01%. The cross-reactivities of the anti-E2 antibody with other steroids were as follows: 17β-estradiol, 100%; estrone, 2.5%; estriol, 0.12%; DHEA, 0.007%; 17α-estradiol, <0.004%; progesterone, <0.004%; testosterone, <0.004%; androstenedione, <0.004%. The cross-reactivities of the anti-testosterone antibody with other steroids were as follows: testosterone, 100%; 5α-dihydrotestosterone, 43.2%; 5α-androstanedione, 33.1%; 5β-androstanedione, 11.4%; 5α-androstan-3α,17β-diol, 9.4%; androstenedione, 0.4%; progesterone, DHEA, and 17β-estradiol, 0,01%; cortisol, <0.001%. After washing the plate with RIA buffer, the standards (5–200 pg/well), the quality-control extract, the test extracts (3.0, 0.8, 1.5, 1.2, and 2.0 mg/well for the analysis of cortisol, DHEA, P4, E2, and testosterone, respectively) and the tracer (hydrocortisone {cortisol [1,2,6,7-3H (N)]-}, DHEA [1,2,6,7-3H (N)], progesterone [1,2,6,7-3H (N)], 17β-estradiol [2,4,6,7-16-17-3H (N)], and testosterone [1,2,6,7-3H (N)]) were added, and the plate was incubated overnight at 4 °C. The bound hormones were separated from the free hormones by decanting and washing the wells in RIA buffer. After the addition of 200 μL of scintillation cocktail, the plate was counted on a β-counter (Top-Count, PerkinElmer Life Sciences Inc., Waltham, MA, USA).

The intra- and inter-assay coefficients of variation were 3.7% and 10.1%, 3.8% and 10.6%, 3.4% and 8.2%, 3.7% and 12.1%, and 4.4% and 11.5% for cortisol, DHEA, P4, E2, and testosterone, respectively. The sensitivities of the assays were 1.23, 0.62, 0.56, 0.77, and 0.33 pg/well for cortisol, DHEA, P4, E2, and testosterone, respectively.

The relationships among hair cortisol, hair DHEA, hair P4, hair E2 and hair testosterone and their respective standard curves, determined through linear regressions, were linear with correlation coefficients of r = 0.99. The models were described by the equations y = 1.147x + 0.943, y = 1.0043x − 0.07, y = 0.969x + 0.751, y = 0.9796x + 1.68, y = 1.023x − 3.76 for cortisol, DHEA, P4, E2 and testosterone, respectively.

### 2.7. Statistical Analysis

The statistical tests were performed using SPSS, version 7.5.21 and R software, version 3.4.0 [33]. The normality of the data distribution was tested using the Shapiro–Wilk test. Non-parametrically distributed data were transformed for parametric testing. The variables were analyzed using a model that considered sex (barrows vs. gilts) and pen as the fixed and random factor, respectively.

## 3. Results and Discussion

The hair hormone concentrations, as affected by sex, are reported in Table 1. Gilts showed higher cortisol (*p* < 0.01) and progesterone (*p* < 0.01) levels and a higher cortisol/DHEA ratio (*p* < 0.01), but they had lower DHEA (*p* < 0.05), 17β-estradiol (*p* < 0.05), and testosterone (*p* < 0.05) levels than barrows. 

The hair hormone concentration is assumed to be a retrospective marker of integrated hormone secretion over longer periods of time [34,35,36,37]. Since animal welfare is of increasing interest for consumers and the wider society, measures providing retrospective information about the quality of the life of the animal during the rearing cycle are becoming increasingly important for the market. For this reason, additional measures are desirable to integrate welfare assessments [38]. The selection of animals involved in the study from the initial 1800 pigs was made by expert technicians in order to have a very homogeneous sample in terms of age and body weight, which was then raised under the same management and in the same environmental conditions. This allowed the samples to be compared. Comparisons, however, were done bearing in mind some characteristics of the bristle, which, as like other hair samples, provides the concentrations of steroids that are referable to a time period of weeks or months, depending on whether the shave–reshave method was used for sampling or not. The hair growth of pigs, as in other species, occurs in cycles (anagen, catagen, and telogen phases). As reported by Mowafy and Cassens [30] and Watson and Moore [31], pig hair follicles pass through one complete cycle per year. The seasons have a marked effect on the activity of hair follicles. The anagen phase is long, about 4 months, and is more present during autumn and winter. For the remaining period and until the end of the summer, the anagen phase is strongly reduced. Molting of the telogen follicles was noticed during the summer months. Hence, the bristles collected in this study at 36 weeks of life had not yet finished their cycle, and the sample collected should be mostly hair in the anagen phase, characterized by maximum hormonal incorporation. Indeed, even though the mechanisms of cortisol incorporation are not yet fully understood, blood-borne steroids can be incorporated into the growing hair shaft when the follicle is connected to the central blood supply [25]. 

In this study, we examined the feasibility and reliability of using hair as a matrix to determine the DHEA and sexual steroid concentrations and the cortisol/DHEA ratio at the end of the pig fattening period.

We found that at 36 months of age, females have significantly higher cortisol levels, significantly lower concentrations of DHEA, and significantly higher cortisol/DHEA ratios than castrated males. 

The differences in cortisol and DHEA concentrations between males and females may be due to numerous influential factors, such as sex, the fact that the females were entire while the males were castrated, different behavioral patterns, body condition, and the metabolism of gonadal steroids. Regarding pig cortisol concentrations, some studies performed in plasma or saliva did not show any differences between castrated barrows and gilts [39,40], while other studies showed higher activity in males than in females [41]. In a study performed in Yucatan miniature swine, Tagliaferro et al. [42] found that DHEA plasma concentrations were lower in females than in entire males, and they supported the hypothesis that DHEA has a biological role in energy balance that, in males, protects against excess adiposity. In fact, DHEA is a steroid hormone that acts at multiple levels. It plays a role in immune system activation [43]; it has anti-inflammatory effects [44] and antioxidant properties [8], and it is involved in lipid metabolism [45]. Pigs affected by stress show an increase in the cortisol level to stimulate metabolism and energy production and a decrease in DHEA [46].

Apart from the environmental changes, such as birth, suckling, weaning, mixing with unfamiliar pigs, movement and handling, growing–finishing and fattening phases [47,48], to which all the animals were subjected, females were also facing puberty. This led to a different setting of the hypothalamic–pituitary–adrenal (HPA) and the hypothalamic–pituitary–gonadal (HPG) axes with consequently different hair concentrations of cortisol and DHEA between castrated barrows and the females. Barrows showed also a significantly lower cortisol/DHEA ratio than females. Both cortisol and DHEA are considered to be indicators of allostasis [5,6] and resilience [7,49], and their ratio is believed to be an index of the anabolic/catabolic balance [50]. This ratio could be an important factor in determining how an individual’s HPA axis is functioning [9,51,52]. Trevisan et al. [23] observed an increased cortisol/DHEA ratio in pigs and suggested that an elevated metabolic effort was needed to cope with the environment in term of housing, feeding, and re-grouping.

Stressors induce both activation of the HPA axis and inactivation of the HPG axis, especially if stress is chronic [46,53]. In the present study, along with cortisol and DHEA, sexual steroids were also evaluated. 

The significantly higher hair P4 concentrations in females than in males may be partly explained by the beginning of ovarian activity, considering that the onset of puberty in gilts occurs at approximately 24–32 weeks of age [54]. The animals were 36 weeks old and, given that hair samples provide a retrospective picture, hair could also have recorded the higher systemic P4 expected in cycling gilts. Consistent with these results, Marchev et al. [55] measured P4 in the blood serum of female pigs and found that its concentration in gilts was significantly higher at the age of 210 days (28 weeks) compared to at the age of 165 days (22 weeks). In males, the P4 increase cannot be related to the onset of gonadal activity, but it most likely may be linked to extra-gonadal production [56,57], which is associated with age.

In our study, the testosterone and E2 hair concentrations were significantly higher in barrows than in females. Robic et al. [58] found that the expression of most genes involved in the biosynthesis of steroids is very similar in the adrenals and testes. Significant amounts of E2 are produced in extra-gonadal tissues, such as the adrenal glands [59], adipose tissue [60], and gastric parietal cells [61]. Studies conducted on castrated goats [62] and on castrated rats [61] showed that the gastrointestinal wall is the main site for the synthesis of aromatase, the enzyme that converts testosterone to E2, and that castration increases the expression of aromatase [62]. Testosterone, an E2 precursor, is self-produced by the gastric parietal cells from androstenedione [63]. Furthermore, Zeng et al. [64] found a detectable serum testosterone level (0.30 ng/mL) in immunized boars at slaughter.

The results obtained in our study were not always in agreement with those emerging from other studies in pigs that used a single-point cortisol measurement, such as in plasma or saliva. This could be due to the use of a cumulative matrix, hair, which is not sensitive to short fluctuations in circulating hormone levels and provides an integrative value of the long-term retrospective circulating steroid hormone concentrations with a single sample; this is useful for detecting long-term activation of the HPA axis.

The use of hair, and particularly of hair not obtained with the shave–reshave method, does not allow us to go back precisely to the type, time, and duration of the exposure to the stressor, but once the threshold values of the various hormones are known, it could retrospectively show that there has been an allostatic load for a considerable time period.

## 4. Conclusions

The use of the trichology matrix collected at the slaughtering could make it possible to obtain interesting indicators of allostatic load and resilience that can be associated with an animal by means of a non-invasive approach that is easy to perform and provides a retrospective assessment.

If future research is able to produce threshold values for the different markers examined, this approach will allow us to evaluate animals under subclinical stress conditions.

## Figures and Tables

**Table 1:** Estimated marginal means of hair hormone concentrations and the cortisol/DHEA ratio recorded for barrows and gilts.

	Gender		SEM	*p*-Value
	Barrows	Gilts		
Cortisol (pg/mg)	4.45	9.06	0.355	<0.001
DHEA (pg/mg)	29.51	17.64	1.554	0.011
Cortisol/DHEA	0.203	0.773	0.048	<0.001
17β-Estradiol (pg/mg)	11.31	3.33	0.864	0.012
Progesterone (pg/mg)	72.60	162.85	8.788	0.009
Testosterone (pg/mg)	8.98	6.92	0.391	0.035

DHEA = dehydroepiandrosterone.
